# Molybdenum and Phosphorus Interact to Constrain Asymbiotic Nitrogen Fixation in Tropical Forests

**DOI:** 10.1371/journal.pone.0033710

**Published:** 2012-03-21

**Authors:** Nina Wurzburger, Jean Philippe Bellenger, Anne M. L. Kraepiel, Lars O. Hedin

**Affiliations:** 1 Department of Ecology and Evolutionary Biology, Princeton University, Princeton, New Jersey, United States of America; 2 Smithsonian Tropical Research Institute, Balboa, Ancón, Republic of Panama; 3 Department of Geosciences, Princeton University, Princeton, New Jersey, United States of America; 4 Department of Chemistry, Princeton University, Princeton, New Jersey, United States of America; DOE Pacific Northwest National Laboratory, United States of America

## Abstract

Biological di-nitrogen fixation (N_2_) is the dominant natural source of new nitrogen to land ecosystems. Phosphorus (P) is thought to limit N_2_ fixation in many tropical soils, yet both molybdenum (Mo) and P are crucial for the nitrogenase reaction (which catalyzes N_2_ conversion to ammonia) and cell growth. We have limited understanding of how and when fixation is constrained by these nutrients in nature. Here we show in tropical forests of lowland Panama that the limiting element on asymbiotic N_2_ fixation shifts along a broad landscape gradient in soil P, where Mo limits fixation in P-rich soils while Mo and P co-limit in P-poor soils. In no circumstance did P alone limit fixation. We provide and experimentally test a mechanism that explains how Mo and P can interact to constrain asymbiotic N_2_ fixation. Fixation is uniformly favored in surface organic soil horizons - a niche characterized by exceedingly low levels of available Mo relative to P. We show that soil organic matter acts to reduce molybdate over phosphate bioavailability, which, in turn, promotes Mo limitation in sites where P is sufficient. Our findings show that asymbiotic N_2_ fixation is constrained by the relative availability and dynamics of Mo and P in soils. This conceptual framework can explain shifts in limitation status across broad landscape gradients in soil fertility and implies that fixation depends on Mo and P in ways that are more complex than previously thought.

## Introduction

Biological di-nitrogen (N_2_) fixation is the major natural source of new N to tropical forests [1] and is thought to influence whether N or phosphorus (P) limits the ability of these forests to respond to CO_2_ fertilization [2], [3]. The macronutrient P has for a long time been thought to limit N_2_ fixation in tropical forests, but some observations question how generalizable this might be. From previous work in Hawaii, Vitousek [4] noted that trace metals might together with P influence asymbiotic N_2_ fixation in soils of a montane tropical forest. Our own studies have demonstrated that molybdenum (Mo) but not P limits asymbiotic fixation in a Panamanian lowland forest [5]. Mo functions as a co-factor in the nitrogenase enzyme (which converts N_2_ to ammonia) while P is needed for ATP and cell growth [6].

Here we examine the influence of both Mo and P on asymbiotic N_2_ fixation in mature lowland tropical forests of Panama. Asymbiotic fixers (bacteria that live freely in soils) are ubiquitous and may contribute substantial atmospheric N_2_ in conditions where symbiotic N_2_-fixers (bacteria that live in association with plant roots) are absent, rare or not fixing (as observed in some mature forests) [7]–[9]. Both asymbiotic and symbiotic fixation contribute to the common pattern of abundance of N relative to P or other plant resources in many tropical forests [7], [10].

Limitation by Mo alone has also been reported for asymbiotic fixers in a highly leached temperate forest soil [11] as well as for symbiotic fixers in some managed pastures and agricultural systems [12], [13]. Responses to trace-metal cocktails combined with P fertilizer in Hawaiian soils [4], [14], [15] also raise the possibility of a Mo influence. Positive responses to P fertilizer [4], [5], [14]–[16] are, however, complicated by the observation that superphosphate fertilizer often contains trace amounts of Mo [5]. In our previous study of a Panamanian forest we found that asymbiotic fixation responded positively to Mo alone, to superphosphate fertilizer in which Mo was a “hidden” contaminant, but not to additions of trace-metal free P [5].

These findings raise fundamental questions about the role of Mo and P in constraining N_2_ fixation: a) Are fixers sensitive to variations in these soil nutrients across landscapes; b) Do fixers respond to both Mo and P in the same location; and c) What conditions and what mechanism(s) favor limitation by one element over the other? These questions identify the need to understand how Mo, P, or both elements together act to constrain N_2_ fixation in nature.

Molybdenum and P are both rock-derived and oxyanions in their bioavailable forms (molybdate, MoO_4_^2−^ and phosphate, PO_4_^3−^ respectively). It is plausible that Mo limitation emerges because molybdate is susceptible to leaching, complexation by organic matter and adsorption to iron (Fe) oxides in highly weathered soils [13], [17]. However, weathered tropical forest soils can also be substantially poor in P [18] and phosphate availability is reduced by sorption and immobilization processes [19]. The remarkable heterogeneity that exists in weathering conditions and distributions of soil nutrients across tropical forests [20] offers further motivation for resolving the dependence of N_2_ fixation on Mo and P across tropical landscapes.

We chose six forest sites distributed along a steep soil P∶Mo gradient in Panamanian lowland forests, across which total soil P spans the range reported from tropical forests world-wide while Mo remains relatively constant. The gradient derives from local variations in geology where andesitic and basaltic lithologies give rise to P-rich soils while marine sediments and rhyolitic tuff produce P-poor soils [21], [22]. The forests are within 8 km of each other, share a similar pool of tree species, and experience a similar climate.

We examined whether limitation on N_2_ fixation depended on landscape-scale variations in the relative abundances of soil Mo and P. We predicted that Mo would limit fixation in P-rich soils while P would limit in P-poor soils. In each forest, we evaluated the response of N_2_ fixation to four nutrient addition treatments: control (C), Mo only (+Mo), trace-metal-free P only (+P) and both elements in combination (+Mo+P). To further understand the mechanism by which nutrient limitation differed across sites we intensively sampled vertical distributions of fixation, Mo and P within each soil and conducted laboratory experiments on the ability of organic matter to interact with bioavailable Mo and P.

## Materials and Methods

### Study Sites

Our study sites are located in mature lowland tropical forests within and south of the Barro Colorado Nature Monument, Republic of Panama. We selected six forests along a steep and well-characterized [21]–[24] gradient in total soil P, from high (AVA and Fairchild) and medium (Gigante) levels on andesitic and basaltic lithologies to low (Barro Verde and Zetek) and very low (Rio Paja) levels on marine sediments and rhyolitic tuff. All forests share a diverse community of tree species, receive 2600 mm annual rainfall, and are subject to a Jan–April dry season. AVA, Fairchild, Barro Verde and Zetek are all located on Barro Colorado Island, while the remaining sites are located on the nearby mainland. All necessary permits were obtained for the described field studies from the National Environmental Authority of Panama (ANAM).

### Soil Nutrient Analysis

We sampled soils down to 80 cm depth from the surface organic layer (O_i_ horizon with little O_e_ and O_a_ development) followed by 10 cm depth increments of mineral soils using an auger or pits at each site. Three replicate samples were homogenized, oven dried (60°C, until stable), and ground by hand in a ceramic mortar and pestle (with liquid N_2_ for organic horizons) to prevent Mo contamination introduced by mechanical grinders made of stainless-steel. Samples were analyzed for total C and N by infrared gas analysis combustion and total Mo and P via microwave digestion (CEM, MARS-5) in trace-metal-free nitric acid and ICP-MS analysis (Thermo-Finnigan, Element 2 at medium resolution). Due to high Fe concentration in tropical mineral soils, digests were diluted and this resulted in a limit of quantification of 0.3 ppm for total Mo in soil. Available Mo and P were extracted from all soils using anion-exchange resin beads. For mineral soils, we conducted extractions (bead∶soil∶water ratio of 1∶5∶60) where beads (Dowex 1×4–200) were free in solution, shaking at 80 rpm for 16 hrs. Beads were separated from soil using a sucrose-density gradient [25], eluted with 10% HNO_3_ [26] and analyzed via ICP-MS. We conducted extractions of O horizon soil samples (bead∶soil∶water ratio of 1∶0.5∶30), using similar methods but with resin beads (Dowex 1×4–50) enclosed in nylon bags. This resin extraction method can be applied to both organic and mineral soils without the confounding effects of altering soil pH or osmotic potential during the extraction procedure. The limit of detection for resin-extractable Mo was 0.25 ppb.

### Nitrogen Fixation Experiments

We followed a method designed and tested within our Panamanian forests for evaluating the response of N_2_ fixation to nutrient amendments [5]. We collected field moist O horizon soils along 100 m transects at each forest site in the late dry season to wet season of 2008 (May–July). Samples were well mixed before nutrient additions and incubations. To each ∼40 g sample (wet weight) we delivered one of four treatments in 14 mls (*n* = 5–15): distilled water (control), 667 µg Mo/kg as Na_2_MoO_4_ (+Mo), +283 mg P/kg as NaH_2_PO_4_ (+P), and 667 µg Mo/kg+283 mg P/kg (+Mo +P). To test that P addition levels were adequate, we also added +2.83, +28.3, +2830 mg P/kg as NaH_2_PO_4_ to soil samples from each site (*n* = 5). Soils were well-mixed after the treatment application, placed in 0.5 L glass jars, and equilibrated outdoors at ambient forest temperature for 10–15 h. To commence acetylene reduction assays (ARA), jars were sealed and 10% of the headspace was replaced with C_2_H_2_. Mixed headspace samples were collected at 5 and 10 h, stored in gas-tight sampling vials and measured within 48 h for C_2_H_4_ on a gas chromatograph (SRI) equipped with a flame-ionization-detector and a Poropak N column. We selected the incubation time based on the physiological response of *Azotobacter* to Mo addition in our own laboratory experiments and to soil communities in the field [27]. In addition, results from our short-term laboratory incubations in all cases matched results from long-term fertilizations in large-scale field plots [5]. Following incubations, we dried and stored samples for moisture and nutrient content. Soil samples from the ARAs were subsequently extracted for available Mo and P content following resin methods described above. Acetylene reduction activity was calculated from the slope of C_2_H_4_ production over two time points and accounting for background levels of C_2_H_4_. Nutrient limitation of N_2_ fixation was tested at each site with a 2-factor ANOVA, with two levels (without addition, with addition) of Mo and P using a general linear model and Tukey *post hoc* means separation tests (all statistical tests evaluated with SAS software, SAS Institute Inc, Cary, NC).

### Experiments on molybdate and phosphate interactions with soil organic matter

We explored how organic matter controls the availability of phosphate and molybdate. First, we compared the fraction of available (i.e., resin-extractable) vs. total (i.e., digestible) P and Mo in O horizon samples across our sites. Second, we added and incubated available forms of Mo (as molybdate; 667 µg Mo/kg as Na_2_MoO_4_; 6-fold ambient) or P (as phosphate; 283 mg P/kg as NaH_2_PO_4_; 0.6-fold ambient) for 20–25 hours in fresh organic soil and then extracted available Mo and P. Third, to verify that soil polyphenols reduce Mo availability, we combined 0.1 mM solutions of molybdenum with increasing concentrations of tannic acid (0–1 mM) and extracted Mo with resin beads using the same methods as above. The quantity of Mo and P that could be recovered in available forms was compared with *t*-tests.

## Results

### N_2_ fixation along a landscape gradient in soil nutrients

**Figure 1:**
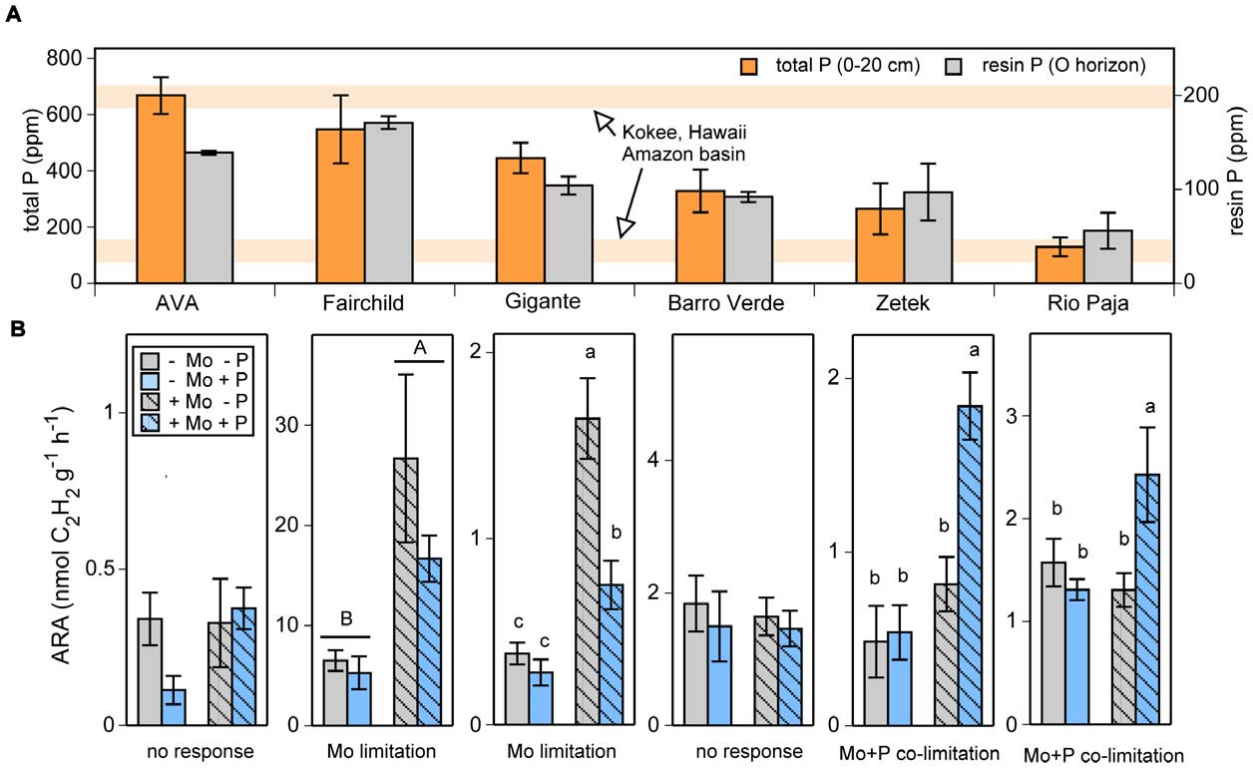
Soil P and the response of nitrogenase activity to nutrient additions from six forest sites arranged from high to low total P. A) Total P (0–20 cm depth; orange bars) and available P (O horizon; grey bars). Pale orange lines represent the range total P levels from montane tropical soils [31] and highly weathered soils in the Amazon basin [20]. B) Nitrogenase activity (acetylene reduction activity (ARA)) in response to nutrient additions: water only (−Mo −P), Mo only (+Mo −P), P only (−Mo +P) and both Mo and P (+Mo +P) in O horizon soil samples (*n* = 5–15). Mo was added as Na_2_MoO_4_ and P was added as NaH_2_PO_4_. Values represent means ± s.e.m. ARA data from Gigante (−Mo −P, +Mo −P and −Mo −P) have been previously reported [5]. Values reported on a mass basis in accordance with convention from global soil data; bulk densities of 0.07 and 0.7–1.2 g/cm^3^ for organic and inorganic horizons respectively can be used to convert to an approximate soil volume basis.

Our six forest sites span a five-fold range of total soil P (top 20 cm of mineral soil)(Fig. 1a). Available P in the O horizon increased three-fold along this gradient in total soil P (r^2^ = 0.84; log-log regression). In contrast, total Mo in mineral soils remained very low across all sites (0.3–0.9 ppm; see methods). In the O horizon both total and available Mo were relatively stable across all sites (Table 1).

**Table 1:** Leaf litter chemistry from six forest sites in lowland Panama arranged from the high to low in total soil P.

	C (%)	N (%)	P (ppm)	Mo (ppb)	Resin Mo (ppb)
AVA	47.3 (0.9)	2.1 (0.1)	437 (73)	144 (16)	0.9 (0.6)
Fairchild	44.4 (0.6)	1.3 (0.05)	629 (40)	131 (17)	0.3*
Gigante	47.9 (0.2)	1.6 (0.1)	384 (24)	70 (13)	2.0 (1.5)
Barro Verde	47.5 (0.8)	1.6 (0.05)	569 (54)	91 (10)	0.9 (0.7)
Zetek	48.8 (0.4)	1.6 (0.1)	377 (51)	86 (11)	1.0 (0.8)
Rio Paja	46.9 (0.2)	1.4 (0.05)	412 (7)	157 (26)	0.3*

Values are means and standard errors in parentheses from each sampling site. Chemical variables included total C and N (%), total digestible P (ppm) and Mo (ppb) and resin extractable Mo (ppb). Many values for resin Mo were at or below the limit of detection (0.25 ppb).

We observed a shift in the response of N_2_ fixers to P and Mo additions across this soil P gradient. In Gigante and Fairchild at the P-rich end of our gradient, nitrogenase activity increased up to four-fold in response to additions of Mo alone and Mo and P combined, but did not respond to P alone (Fig. 1b)(*F*_1,3_ = 36, *p*<0.0001 for Gigante, *F*_1,3_ = 16, *p*<0.001 for Fairchild, analysis of variance (ANOVA). In contrast, in Zetek and Rio Paja at the P-poor end of our gradient, nitrogenase activity increased 1.5 to 4 times only in response to the combined addition of Mo and P, but not to either element applied individually (Fig. 1b)(Mo by P interaction, *F*_1,3_ = 5.7, *p* = 0.023 for Zetek and *F*_1,3_ = 1.7, *p* = 0.039 for Rio Paja, ANOVA). At AVA and Barro Verde we observed low to medium rates of ambient N_2_ fixation but no clear response to nutrient amendments. To verify that we added sufficient available P, we dosed soils with phosphate at three additional levels (in total spanning four orders of magnitude) but observed no positive response in any forest (Table 2).

**Table 2:** Nitrogenase activity (Acetylene reduction activity in nmol C_2_H_2_ g^−1^ h^−1^) in response to additions of P to soils from six forest sites (mean (s.e.m.); *n* = 5.

		P addition (mg/kg)		
Site	control	+2.83	+28.3	+2830
AVA	0.34 (0.08)	0.24 (0.06)	0.11 (0.04)	0.22 (0.04)
Fairchild	6.5 (1.04)	5.00 (1.33)	5.29 (1.64)	5.64 (1.62)
Gigante	0.38 (0.06)	0.17 (0.03)	0.28 (0.07)	0.24 (0.04)
Barro Verde	1.84 (0.42)	1.40 (0.42)	1.20 (0.53)	0.92 (0.33)
Zetek	0.48 (0.21)	0.59 (0.10)	0.54 (0.16)	0.36 (0.05)
Rio Paja	1.57 (0.23)	1.85 (0.29)	1.52 (0.14)	1.10 (0.07)

### N_2_ fixation and soil chemistry with depth

**Figure 2:**
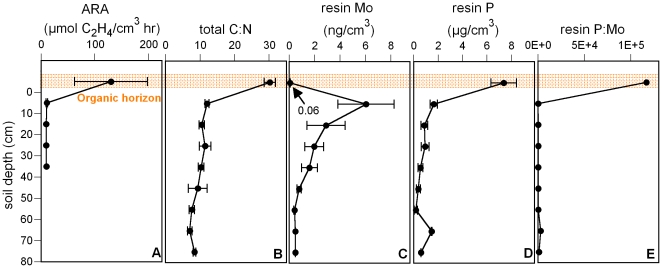
Vertical soil gradients in nitrogenase activity and nutrient abundance averaged across six tropical forests. A) Nitrogenase activity (acetylene reduction activity, ARA). B) Total C to N mass ratio. C) Available (resin-extractable) Mo. D) Available (resin-extractable) P. E) Resin-extractable P to Mo mass ratio. Uppermost soil layer is the organic soil horizon, mineral soil begins at 0 cm. Values (mean ± s.e.m.) represent measurements from six tropical forest sites. Values reported on a soil volume soil to reflect what microbial communities experience, and to minimize the confounding influence of differences in density between organic and inorganic horizons.

Individual soil profiles across all our study sites displayed similar and clearly delineated vertical gradients in N_2_ fixation, mass ratios of total carbon (C) to N and of available Mo and P (Fig. 2). The highest rates of N_2_ fixation always occurred in the upper soil horizon (O), which was rich in organic matter but poor in total N (C∶N ratios ∼30)(Fig. 2a). Deeper soil layers displayed negligible fixation, low C content, and abundant N relative to C (C∶N ∼10). Available Mo was exceptionally low in the organic soil layer, peaked in the upper mineral soil horizon and declined with soil depth. In contrast, available P was greatest in the organic soil horizon and declined steadily with depth. As a result the upper organic soil horizon was characterized by ratios of available P∶Mo that were up to five orders of magnitude greater than those of deeper soil layers (Fig. 2e).

### Experiments on molybdate and phosphate availability

**Figure 3:**
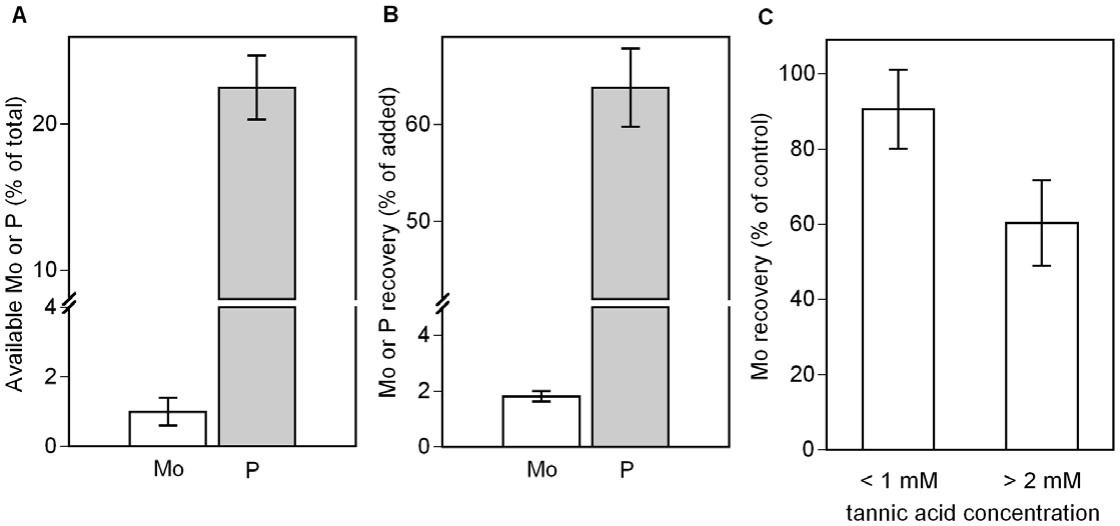
Available Mo and P in organic soils and Mo recovery from tannic acid solutions. A) Fraction of available (resin-extractable) Mo and P in O horizon soils averaged across six forest sites. B) Recovery of Mo and P (resin-extractable) after addition and 20–25 hours of incubation of molybdate and phosphate to freshly sampled O horizon soils of six forest sites. C) Recovery of Mo (resin-extractable) from 0.1 mM solutions of Mo and increasing concentrations of tannic acid (*n* = 6) relative to tannic-acid free controls. Values represent means ± s.e.m.

While high available P has been reported from the O horizon of P-poor tropical soils [28]–[30], we explored whether interactions with soil organic matter could explain the occurrence of high P∶Mo ratios in Panamanian organic soil horizons. First, we found that available Mo constitutes <2% of total Mo in O horizon samples across our sites, while available P accounts for >20% of total P (Fig. 3a)(*p*<0.0001, *t*-test). Second, after experimental additions of molybdate and phosphate we could recover only very little (<2%) of newly added Mo from the soil matrix in the form of available Mo. In contrast, we could recover substantial amounts (>60%) of newly added P in the form of available P (*p*<0.0001; *t*-test; Fig. 3b). Third, to verify that soil polyphenols act to reduce Mo availability, we combined solutions of molybdate and tannic acid in the laboratory and observed that the recovery of available Mo decreased with increasing concentrations of tannic acid (Fig. 3c).

## Discussion

Soils from our sites in lowland Panama span the range of total P content reported from tropical forests on well-developed soils world-wide (Fig. 1a): from P-rich montane soils [31] (similar to our AVA site) to large areas of P-deficient soils in lowland Amazonia [20] (similar to our Rio Paja site). In contrast, total soil Mo was low compared to the few measures available from natural soils [32] and did not vary systematically across our sites. Therefore, our gradient primarily captures variations in P relative to Mo and provides a localized experimental system for understanding how P and Mo might interact to constrain N_2_ fixation in tropical landscapes.

Our vertical sampling of soil profiles revealed that asymbiotic fixation primarily occurred in the O-horizon across all our sites [4], [5], [15], [33]. We infer that asymbiotic N_2_-fixers are stimulated in the O horizon by the continual input of energy-rich plant litter that is poor in N (as indicated by high C∶N ratios) relative to the physiological demand of soil microbes. Although a suite of environmental factors (e.g., moisture, temperature) can influence the rate of fixation, our goal here was to examine the specific role of Mo and P in limiting fixation in the O horizon of a given soil.

We predicted that Mo would limit N_2_ fixation in P-rich soils while P would limit in P-poor soils. Nutrient amendments stimulated N_2_ fixation substantially in four of our six sites following a clear pattern: fixation was limited by Mo alone in P-rich soils but co-limited by both Mo and P in P-poor soils. Contrary to our expectation, we never found exclusive P limitation at any of our sites. The influence of Mo extended even to the most P-poor sites, where additions of both Mo and P were necessary to stimulate rates of fixation (Zetek and Rio Paja in Fig. 1b). We did not observe statistically significant responses to nutrient additions in two locations, perhaps due to the influence of edaphic factors other than Mo and P [34] as suggested by the low fixation rates observed at the AVA site. The slightly weaker response to combined Mo and P addition at Gigante was possibly caused by interference of the macronutrient phosphate on the availability of the micronutrient molybdate.

Our results offer a direct experimental demonstration that asymbiotic N_2_-fixers can be influenced by broad variations in Mo and P availability across forests, and that the influence of Mo can extend (either alone in P-rich soils or in combination with P in P-poor soils) across soil P levels. These findings, together with the lack of P-only limitation across our P gradient raise important questions about how Mo and P interact to constrain fixation in tropical soils.

We next examined why Mo was such a prevalent constraint on fixation in the organic soil layer across our sites. It may be reasonable to expect that molybdate and phosphate behave similarly in soils as they are both oxyanions. However, we found that available forms of Mo and P possessed dramatically different trends with soil depth. Most strikingly, the organic horizon possessed high levels of available P relative to Mo and was characterized by available P∶Mo ratios that were up to five orders of magnitude greater than those of deeper soil layers. These contrasting distributions of Mo and P with soil depth create a localized biogeochemical niche (the O horizon) in which high organic matter, high fixation rates, and high available P to Mo ratios could lead to Mo limitation on N_2_ fixation.

We next examined whether differences in how Mo and P interact with the plant-soil organic matter cycle can explain whether Mo and/or P emerge as primary constraints on N_2_ fixation. Mo is one of the least abundant plant essential elements [35] and is generally scarce relative to P in the organic horizon. For example the mass ratio of total digestible P∶Mo averaged ∼5000 in O horizons across our study sites, which is 6–7 times greater than the optimal P∶Mo ratio required by free-living N_2_-fixing microorganisms in our laboratory cultures (680–850 for *Azotobacter vinelandii*; [27]). This discrepancy was even further magnified when considering available forms of P and Mo, such that P∶Mo ratios exceeded the cellular ratio of N_2_-fixers by up to 200 times in the O horizon. These observations offer direct evidence that the availability of P can be abundant relative to Mo in the organic soil layer.

We considered the possibility that the high ratio of available P∶Mo in the O horizon is caused by differences in how each element interacts with organic matter. Mo is held strongly by polyphenolic complexes that can diminish bioavailability in soils [17], while most organic P can be hydrolyzed by extracellular phosphatases produced by plants and microbes [36], [37]. We found support for the hypothesis that plant organic matter preferentially reduces the availability of Mo relative to P. Not only does a large fraction of P exist in highly available forms relative to Mo, but additions of Mo and P showed preferential binding of molybdate to natural organic matter and to experimental additions of tannic acid. These results indicate that relative to phosphate, free molybdate is almost entirely consumed by soil even at concentrations much higher than ambient levels.

We speculate that what little molybdate is free in solution in the soil organic layer is likely consumed by biological uptake or lost by leaching to deeper soil horizons. In addition, some organically-complexed Mo can be accessed by free-living N_2_-fixers that have evolved high-affinity systems for Mo uptake [17], [38]. The extent of such mining for Mo is poorly known and may depend on the energetic cost and effectiveness of metallophore production in soil environments. The presence of such a specialized uptake mechanism, however, indicates that Mo-poor environments have acted as a strong constraint on asymbiotic N_2_-fixing organisms over evolutionary time.

We identify the existence of broad gradients in nutrient limitation on N_2_ fixation across tropical forests. Our findings are consistent with the following set of interacting mechanisms: 1) N_2_ fixation is favored in the organic soil horizon because the plant-soil cycle creates a vertical gradient in energy-rich and N-poor organic matter; 2) In this specialized niche Mo is complexed strongly while most organic P is readily turned over, favoring the availability of P over Mo and making possible the localized emergence of Mo over P limitation on N_2_-fixers; and 3) N_2_-fixers respond to broad trends in soil P availability, such that Mo constrains fixation in P-rich soils while Mo and P together constrain fixation in P-poor soils.

Our results offer a link between Mo and P stoichiometry from the scale of molecular biochemistry to that of landscape nutrient cycles. Sole Mo-limitation indicates deficiency in the FeMo co-factor of nitrogenase [39], while Mo+P co-limitation suggests deficiency in both the co-factor and the P-rich molecules (e.g., ATP, RNA and phospholipids) that support N_2_ fixation and cell growth [6]. These constraints may, in turn, scale up to larger levels of observation if Mo and P interact across soils in general (as we found in lowland Panama).

These results lend support to the idea of an emerging paradigm shift in the way we view nutrient limitation in tropical forests. Our findings suggest that N_2_ fixation should not be considered solely constrained by P in conceptual theories or global biogeochemical models (e.g., [40]). The idea of uniform single-element limitation is increasingly challenged as more complex relationships between nutrient cycles emerge. In these Panamanian forests, this complexity is a consequence of the presence of N_2_ fixing organisms, inputs of P and Mo (from weathering and dust), and interactions of C, N, P and Mo within different soil layers. It is critical to determine if the soil-based mechanisms observed in this study are pervasive across land ecosystems where N_2_ fixation is an important source of N.

Our conceptual model may also apply to N-poor ecosystems in temperate and boreal biomes that have highly leached soils (thereby low Mo availability), distinct organic horizons (thereby high Mo complexation) and where asymbiotic N_2_ fixation can be the major vector of new N inputs. A previous experiment in temperate rainforests identified a direct influence of Mo on fixation [11], but there exists little to no information on how Mo and P interact across soils of extratropical as well as tropical biomes.

A critical issue is whether our results extend also to symbiotic N_2_-fixers. Limitation by Mo has to our knowledge not been experimentally evaluated for symbiotic fixers in natural communities including tropical forests. It is plausible that the new concept of Mo+P co- limitation also applies to symbiotic fixers that primarily access nutrients from organic-rich soil horizons. Our findings therefore raise the question of how symbiotic N_2_-fixing plants relate to variations in Mo and P distributions in soils.

Here we offer a new mechanistic model against which the diverse responses of tropical N_2_ fixation to nutrient additions (including superphosphate fertilizer) can be interpreted [4], [5], [14]–[16]. We demonstrate that Mo and P act together to limit fixation across a range of soil P concentrations, from among the highest to the lowest reported for tropical forest ecosystems. In no case did we observe sole P limitation, but this constraint might emerge in soils where Mo is more abundant than it is across our gradient. Re-examining previous studies with our current hindsight is challenging, but we note that the common use of superphosphate fertilizer might confound the very interactions of P and Mo that our present study has uncovered. Since tropical soils vary greatly in distributions of macro- and micronutrients [20] it is likely that the land N cycle depends on P and Mo in ways that are more complex than we have previously considered.
